# Urolithin B suppressed osteoclast activation and reduced bone loss of osteoporosis via inhibiting ERK/NF‐κB pathway

**DOI:** 10.1111/cpr.13291

**Published:** 2022-06-16

**Authors:** Yajun Li, Qi Zhuang, Lihong Tao, Kai Zheng, Shuangshuang Chen, Yunshang Yang, Chengcheng Feng, Zhifang Wang, Haiwei Shi, Jiandong Shi, Yiling Fang, Long Xiao, Dechun Geng, Zhirong Wang

**Affiliations:** ^1^ Translational Medical Innovation Center Zhangjiagang TCM Hospital Affiliated to Nanjing University of Chinese Medicine Zhangjiagang China; ^2^ Department of Orthopedics Zhangjiagang TCM Hospital Affiliated to Nanjing University of Chinese Medicine Zhangjiagang China; ^3^ Department of Rheumatology Zhangjiagang TCM Hospital Affiliated to Nanjing University of Chinese Medicine Zhangjiagang China; ^4^ Department of Orthopedics The First Affiliated Hospital of Soochow University Suzhou China; ^5^ Department of General Practice, The First People's Hospital of Zhangjiagang Soochow University Zhangjiagang China

## Abstract

**Objectives:**

The main target of current drugs for alleviating bone loss is osteoclasts. However, the long‐term application of such drugs will also cause side effects. Therefore, it is of great need to develop new and safer therapeutics for osteoporosis. In recent years, drug development based on gut microbiota has gradually attracted attention. This manuscript investigates the inhibitory effect of urolithin B (UB) on osteoclastogenesis and differentiation in vitro and in ovariectomized (OVX) mice.

**Materials and Methods:**

CCK‐8 was used to analyse the cytotoxicity of UB; BMMs cells were differentiated into osteoclasts by RANKL, and respectively treated with 1, 5, and 25 μmol/L UB during this process. After one week of intervention, tartrate‐resistant acid phosphatase (TRAP) staining was used to analyse the number and average area of osteoclasts. F‐actin staining and immunofluorescence staining were conducted to evaluate the morphology and function of osteoclasts. Bone resorption function of osteoclasts was detected by Pit Formation Assay. The expression of osteoclast‐related protein genes in RAW264.7 cells were investigated via western blot and RT‐PCR assays. Western blot analysis of RANKL‐mediated activation of MAPK/NF‐κB pathway after 0, 5, 15, 30, 60 min of intervention. For in vivo experiments, OVX mice received intraperitoneal injection of 10, 50 mg/kg every two days, 8 weeks later, the femurs of mice were taken for morphological analysis, and the serum content of CTX‐1, a bone metabolism index, was analysed.

**Results:**

UB could inhibit the osteoclast differentiation of rankl‐induced bone marrow macrophages (BMMs) and RAW264.7 cells in vitro, suppress the uptake activity of hydroxyapatite and expression of osteoclast‐related gene MMP9, CTSK, NFATc1 and c‐fos. Furthermore, UB repressed the rankl‐induced phosphorylation and degradation of IκB and the phosphorylation of P65 in the NF‐κB pathway of RAW264.7 cells, and also down‐regulated the phosphorylation level of ERK in the MAPK pathway. For in vivo studies, UB‐treated OVX mice showed more significant improved various parameters of distal femur compared with the control group, with fewer NFATc1, MMP9 and TRAP‐positive osteoclasts in bone tissues, and less serum content of CTX‐1.

**Conclusion:**

Urolithin B attenuated bone loss in OVX mice by inhibiting the formation and activation of osteoclasts via down‐regulation of the ERK/NF‐κB signalling pathway.

## Graphical abstract

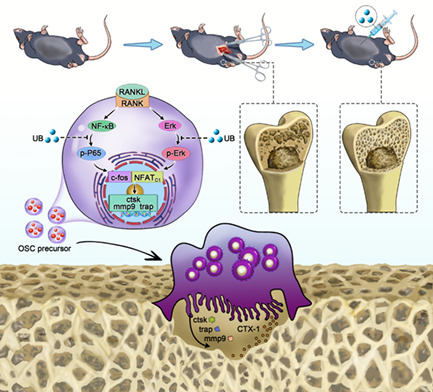

## 1 INTRODUCTION

Osteoporosis is an abnormal bone homeostasis caused by various primary or secondary factors,^1^, ^2^, ^3^ with microscopic behaviours at abnormally activated bone resorption activity of osteoclasts leading to negative imbalance of bone remodelling.^4^ Osteoclasts are multinucleated giant cells derived from monocyte/macrophage precursors of bone marrow haematopoietic stem cells. Currently, the main drugs for the clinical treatment of osteoporosis are bisphosphonates, teriparatide, denosumab, etc.^5^ However, long‐term drug therapy will also cause a series of complications.^6^, ^7^, ^8^ There, it is necessary to develop a strategy that can safely and effectively inhibit the abnormal activation of osteoclasts.

In recent years, drug development based on gut microbiota has gradually attracted attention.^9^, ^10^ Urolithin B (UB) is a product of ellagitannins, which are abundant in pomegranate, walnut and berries, transformed by intestinal microflora,^11^, ^12^ and has anti‐inflammatory and antioxidant properties,^13^ anti‐cancer^14^ and other biological activities. It can effectively protect the intestine and improve intestinal immune function.^15^ Huang et al. found that UB can reduce the occurrence of myocardial arrhythmia after hypoxia through p65 nuclear translocation,^16^ and Chen et al. showed that UB can effectively alleviate cognitive defects and improve the conditions related to brain aging through MAPK pathway.^17^ Ellagine tannin and ellagic acid are the last products of UB in the intestine. It has been reported that the ellagitannin and its hydrolysate ellagic acid can alleviate osteoporosis in ovariectomy (OVX) mice by inhibiting the differentiation of osteoclasts.^18^, ^19^ However, due to its poor water solubility, the serum drug concentration does not increase with increased dosage.^20^ Thus, cells of tissues and organs in the body may not directly interact with ellagitannins and ellagic acid. In contrast, it is widely reported that UB and its derivatives can reach the plasma concentration of micromolar level^21^ and exert biological activities *in vivo*,^22^, ^23^, ^24^, ^25^ revealing that UB has higher bioavailability than ellagitannin and ellagic acid.^21^

In this study, the effect of UB on osteoclast differentiation induced by bone marrow‐derived macrophages (BMMs) and RAW264.7 cells in vitro was investigated. And OVX model mice was established to study the therapeutic efficacy of UB on osteoporosis. Furthermore, the possible mechanisms of UB inhibiting osteoclast differentiation were explored.

## 2 MATERIALS AND METHODS

### 2.1 Cell culture

Cells were cultured at 37°C incubator with humidified environment and 5% CO_2_. RAW264.7 cells (FH0328, FuHeng BioLogy, ShangHai, China) were incubated and passaged with DMEM medium (SH30022.01, Cytiva, Pittsburgh, USA) containing 10% FBS (16,140,071, Gibco, Rockville, USA) and 100 μ/mL penicillin–streptomycin‐amphotericin B (C100C8, NCM Biotech, SuZhou, China) before seeding and drug intervention, and the medium was changed every other day. The BMMs were obtained from the fresh femurs of 6‐week‐old C57/BL6 mice as follows. First, the mice sacrificed by anaesthesia were sterilized in 75% ethanol for 5 min. Then the soft tissues of both lower extremities were cleaned, and the femur and tibia were separated rinsed twice with DMEM medium. Next, cut the bone ends with surgical scissors in the ultra‐clean bench, rinse the bone marrow cavity with DMEM and filter it through a 70 μm cell strainer. And add the red blood cell lysate and centrifuge at 1000 r/min for 5 min to remove the supernatant, finally resuspend and culture in DMEM medium with 10% FBS, 50 ng/mL M‐CSF (462‐TEC‐010/CF, R&D Systems, Minneapolis, MN, USA) and 100 μ/mL Penicillin–Streptomycin‐Amphotericin B.

### 2.2 Cell viability assay

The cytotoxicity of UB (SML1649, Sigma‐Aldrich, Missouri, USA) was detected by CCK‐8 assay. BMMs or RAW264.7 cells were seeded at a density of 1 × 10^4^/well in 96‐well plates for 3–4 h, and then intervened with different concentrations of UB (0, 1, 5, 25, 50, 100, 150 μmol/L) for 1, 2 or 3 days, followed by addition of 10 μl of CCK‐8 buffer (C0038, Beyotime, Shanghai, China) to each well and incubation at 37°C for another 1 h. Then the absorbance at 450 nm was measured using a microplate reader (BioTek, Vermont, USA).

### 2.3 Tartrate‐resistant acid phosphatase (TRAP) staining

1 × 10^5^/well BMMs were seeded in 12‐well plates, co‐incubated with 50 ng/mL M‐CSF and 50 ng/mL RANKL, and then respectively treated with 0, 1, 5, 25 μmol/L UB for 5 days. The cells were washed twice with PBS for 15 min, fixed with paraformaldehyde, stained with a TRAP staining kit (387A‐1KT, Sigma) and finally photographed. The average area of osteoclasts (cells with more than three nuclei) per well was measured using ImageJ software (NIH, Bethesda, Maryland, USA).

### 2.4 Immunofluorescence staining assay

BMMs were cultured in a 12‐well plate at a density of 1 × 10^5^/well and intervened with 50 ng/mL RANKL, 50 ng/mL M‐CSF and different concentrations of UB (0, 1, 5, 25 μmol/L) for one week. Then osteoclasts were sequentially washed 3 times with PBS, fixed with 4% paraformaldehyde for 30 min, and permeabilized with Triton X‐100 for 10 min. Next, cells were incubated with primary anti‐MMP9 antibody (1:1000, ab38898, Abcam, Cambridge, UK) at 4°C overnight, followed by being stained with Alexa Fluor 555 and Molecular Probes Alexa Fluor 488 Phalloidin (1:1000, #8878 s Cell Signalling Technology, Danvers, USA) for 1 h in the dark. After 10 min fixation with DAPI‐containing mounting fluid, the cells were imaged via EVOS M5000 cell imaging system (Thermo Fisher Scientific, Bothell, WA, USA) to quantify area and the number of nuclei per osteoclast.

### 2.5 Pit formation assay

1 × 10^5^/well of BMMS were seeded into 24‐well osteo‐assay plates (3987, Corning, ME, USA) and treated with different concentration of UB (0, 1, 5, 25 μmol/L), 50 ng/ML M‐CSF, and 50 ng/ML RANKL. 5 days later, the images of the bone resorption area were recorded with a microscope and quantitatively analysed by IMAGEJ software

### 2.6 Western blot analysis

1 × 10^5^/well RAW264.7 cells were seeded into a 6‐well plate and adhered to the wall, then treated with medium containing 50 ng/mL RANKL and different concentrations of UB (0, 1, 5, 25 μmol/L). Then the total protein of adherent cells was extracted by adding the lysis buffer and the protein content was determined. Protein samples were separated by electrophoresis and then transferred to PVDF membranes (IPVH00010, Millipore Corporation, Billerica, USA), which were blocked with blocking solution for 15 min and incubated with primary antibodies overnight at 4°C. The specific pathway inhibitors used are as follows: SCH772984 (A12824, Adooq Bioscience, Irvine, USA), SC75741 (A14278, Adooq Bioscience). The primary antibodies used are as follows: Osteoclast‐related functional proteins: MMP9 (1:1000, ab38898, Abcam), CTSK (1:1000, ab19027, Abcam), transcription factor: c‐Fos (1:1000, ab190289, Abcam), NFATc1 (1:1000, ab25916, Abcam), and MAPK, NF‐κB pathway‐related protein antibodies: JNK (1:1000, ab179461, Abcam), p‐JNK(Thr183/Tyr185) (1:1000, #4668, CST), and p38 (1:1000, ab170099, Abcam), p‐p38(Thr180/Tyr182) (1:1000, #4511, CST, Boston, USA), ERK (1:1000, #4695, CST), p‐ERK(Thr202/Tyr204) (1:1000, #4377, CST), IκB‐α (1:1000, ab32518, abcam), p‐IκBα(Ser32) (1:1000, #2859, CST), P65 (1:1000, ab16502, Abcam), p‐P65(Ser536) (1:1000, #3031, CST). After washing with TBST (BP‐G0004, CWBiotech, Beijing, China), membranes were incubated with secondary antibodies for 2 h and observed with the chemiluminescent HRP substrate (WBKLS0500, Millipore Corporation).

### 2.7 Quantitative RT‐PCR analysis

1 × 10^5^/well BMMs were cultured in 6‐well plates with DMEM medium containing 50 ng/mL M‐CSF, 10% FBS, 100 μ/mL penicillin–streptomycin‐amphoteric acid B and 50 ng/mL RANKL. Then BMMs were treated with 0, 1, 5, and 25 μM of UB, respectively. Two days later, total RNA was extracted from cells using an RNA rapid extraction kit (RN001, Yishan Biotechnology, ShangHai, China), and reverse‐transcribed to cDNA via a Reverse Transcription Kit (G898, ABM, Vancouver, Canada). Then, real‐time quantitative PCR was performed using SYBR Green PCR MasterMix (A46110, Applied Biosystems， Vilnius, Lithuania). The PCR cycle conditions were set as: 94°C for 10 min, 95°C for 15 s, and 60°C for 60s of 40 cycles, and 4 replicate wells were set for all reactions to ensure the accuracy of the data. The qPCR primers utilized were shown in (Table 1).

**TABLE 1:** Primers used in RT‐PCR

Gene	Forward pssrimer (5′ → 3′)	Reverse primer (5′ → 3′)	Size
[Traditional RT‐PCR]			
NFATc1	5′‐GACCCGGAGTTCGACTTCG‐3′	5′‐TGACACTAGGGGACACATAACTG‐3′	97 bp
CTSK	5′‐GAAGAAGACTCACCAGAAGCAG‐3	5′‐TCCAGGTTATGGGCAGAGATT‐3′	102 bp
c‐fos	5′‐CGGGTTTCAACGCCGACTA‐3′	5′‐TTGGCACTAGAGACGGACAGA‐3′	166 bp
TRAP	5′‐CACTCCCACCCTGAGATTTGT‐3′	5′‐CATCGTCTGCACGGTTCTG′	118 bp
V‐ATPase	5′‐CAGAGCTGTACTTCAATGTGGAC‐3′	5′‐AGGTCTCACACTGCACTAGGT‐3′;	111 bp
MMP9	5′‐CTGGACAGCCAGACACTAAAG‐3′	5′‐CTCGCGGCAAGTCTTCAGAG‐3′;	145 bp
GAPDH	5′‐AGGTCGGTGTGAACGGATTTG‐3′	5′‐TGTAGACCATGTAGTTGAGGTCA‐3′	123 bp

### 2.8 OVX‐induced osteoporosis mouse model

The animal experiments in this study were approved by the Animal Ethics and Welfare Committee (AEWC) of Zhangjiagang TCM Hospital Affiliated to Nanjing University of Chinese Medicine (Approval date: 2021‐06‐10, Approval NO: AEWC‐20210610). A total number of 40 C57BL/6 mice aged six weeks with an average weight of 20 g were purchased from the JOINN laboratory in Suzhou, China (NO: 202114550). Mice were randomly divided into 4 groups: sham‐operated group, OVX group, OVX + low‐dose UB group, and OVX + high‐dose UB group. The mice in the OVX group and the OVX + UB groups were excised under anaesthesia and the incisions were sutured after the bilateral ovaries and surrounding adipose tissues were removed. In the sham operation group, only the ovaries were exposed, and the surrounding adipose tissues were removed and then incorporated into the abdominal cavity to suture the incisions. Three weeks later, the OVX + low‐dose UB group was intraperitoneally (i.p.) injected with 10 mg/kg UB every two days, while the UB dosage of OVX + high‐dose UB group was 50 mg/kg.^17^, ^26^ In contrast, the sham group and OVX group were treated with saline. After 8 weeks of intervention, we took blood from the orbit, centrifuged the upper serum, took out the femur and fixed it in 4% paraformaldehyde.

### 2.9 ELISA experiment

Mouse cross‐linked C‐telopeptide of type I collagen (CTX‐1) ELISA kit (M3023, Elabscience, Wuhan, China) was used to analyse the content of bone metabolism marker CTX‐1 in serum according to ELISA instructions. First, 100 μl of standard substance and tested samples were respectively added into the plate wells, and incubated at 37°C for 90 min. Then the liquid was discarded, and 100 μl of biotinylated antibody working solution was loaded and incubated at 37°C for 60 min. After removal of the liquid and washing for three times, 100 μl of HRP‐conjugated working solution was added and incubate at 37°C for 30 min. Discard the solution, repeat the washing process for 5 times, add 90 μl of substrate solution to each well and incubate at 37°C for about 15 min. Finally, supplement 50 μl of stop solution per well and measure the absorbance (OD value) with a microplate reader at a wavelength of 450 nm for calculation of sample concentration. The blank well was set as zero.

### 2.10 Micro‐CT scanning

μCT images of the left femurs of four groups (*n* = 5 in each group) were obtained using a SkyScan1176 high‐resolution microcomputed tomography scanner (SkyScan, Knotich, Belgium). The scanning parameters were set as 9 μm per layer, 80 kV voltage, and 100 mA current. The processed 3D images were then imported into CTAn software (Brukermicro‐CT, Kontich, Belgium) to measure and analyse the bone mineral density (BMD, g/cm3), bone surface (BS, mm^2^), bone volume (BV, mm^3^), bone surface/ total volume (BS/TV, 1/mm), bone volume/total volume (BV/TV, %), trabecular separation/Spacing (Tb. Sp, mm), and number of trabecular bone (Tb. N, 1/mm) of the distal femurs.

### 2.11 Histological analysis

Histological analyses were performed after the completion of the Micro‐CT scanning. The femurs were decalcified with 10% EDTA for 21 days, then the distal femurs were embedded in paraffin and sliced into 5 μm thick sections using a microtome. Sections were then stained with haematoxylin and eosin (H&E), TRAP.

For H&E staining, after dewaxing and rehydrating, the sections were stained with haematoxylin solution for 10 min, rinsed with running water until the colour turned blue, followed by incubation with 0.5% eosin staining solution for 5 min. Next, sections were subsequentially dehydrated in 95% ethanol and anhydrous ethanol for 3 min, and washed twice with xylene for 5 min, and finally air‐dried and sealed.

For TRAP staining procedure, first, deparaffinize and rehydrate the sections, put them in the TRAP solutions which was prepared via TRAP staining kit in the dark at 37°C for 50 min, then rinse with distilled water and counterstain with haematoxylin for 5 min. Then, 95% and 100% gradient dehydration were conducted and followed by xylen transparent for 5 min and sealed after air‐drying.

### 2.12 Immunohistochemical analysis

IHC staining was performed according to the Enhance Labelled Polymer System (ELPS) method. First, deparaffinize and rehydrate the sections, followed by incubation with 100 μl hydrogen peroxide blocking solution for 10 min. After washing three times with PBS for 5 min each, 100 μl 5% BSA blocking solution was applied to block the sections for 20 min, then 50 μl primary antibodies such as MMP9 (PA5‐13199, Invitrogen, Carlsbad, USA) or NFATc1 (A1539, Abclonal, Wuhan, China) was added to incubate for 12 h. Next, the sections were washed with PBS for another 3 times, 5 min each, incubated with 50 μl of secondary antibodies for 30 min and washed with PBS three times for 5 min each. Then, DAB was used for colour development and after rinsing with running water, sections were counterstained with haematoxylin for 30 s and rinsed again with running water for 5 min. And after being differentiated with hydrochloric acid alcohol for 1 s and rinsed with for 10 min to turn blue. The gradient dehydration was repeated with 95% ethanol and anhydrous ethanol for 5 min, followed by being soaked in xylene for three times with 5 min each and finally mounted. Stained sections were all photographed using a Nikon Eclipse Ci‐L light microscope (Nikon, Tokyo, Japan). Histomorphological analysis of bones was performed via Panorama Histocytometry Quantitative Analysis System (TissueFAXS Plus, TissueGnostics GmbH, Austria).

### 2.13 Statistical analysis

SPSS 25.0 software was used for statistical analysis in this study. All experimental data are expressed as mean ± standard deviation (*SD*). The t test was used to compare the two groups, and the one‐way analysis of variance (ANOVA) was applied for data comparison of more than two groups. *p* < 0.05 indicated that the difference was statistically significant.

## 3 RESULTS

### 3.1 UB inhibited RANKL‐induced osteoclast differentiation

**FIGURE 1:**
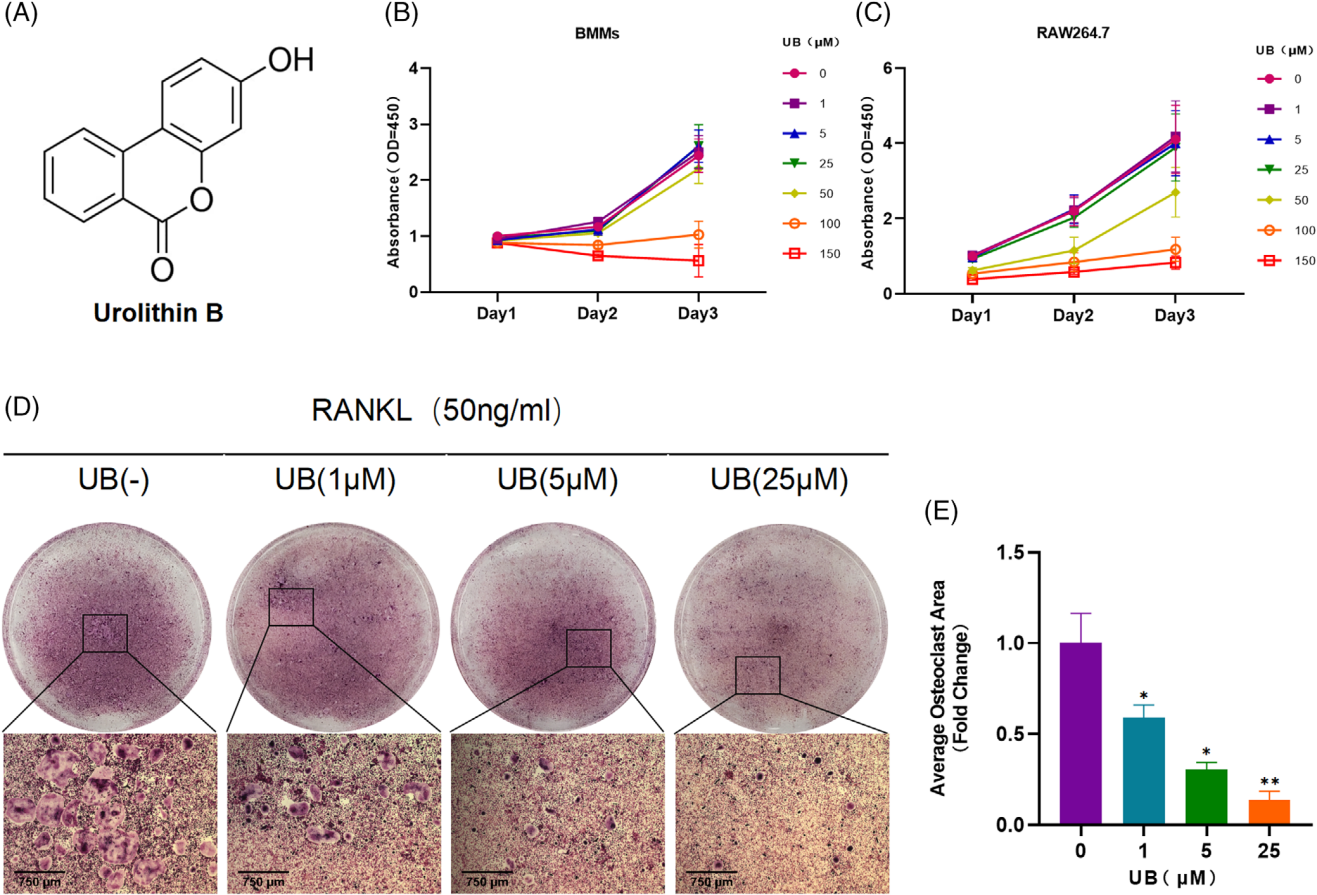
Urolithin B inhibits RANKL induced osteoclast formation in vitro. (A) The structure of urolithin B. (B‐C) CCK‐8 assay was performed to evaluate bmms cells and RAW264.7 cell viability. (D) Representative images of in vitro TRAP staining. (E) Quantify the area of trap osteoclasts in each group. *n* = 3 per group; Scale = 750 μm；*p < 0.05, **p < 0.01 vs. the RANKL‐induced group (without UB treatment)

**FIGURE 2:**
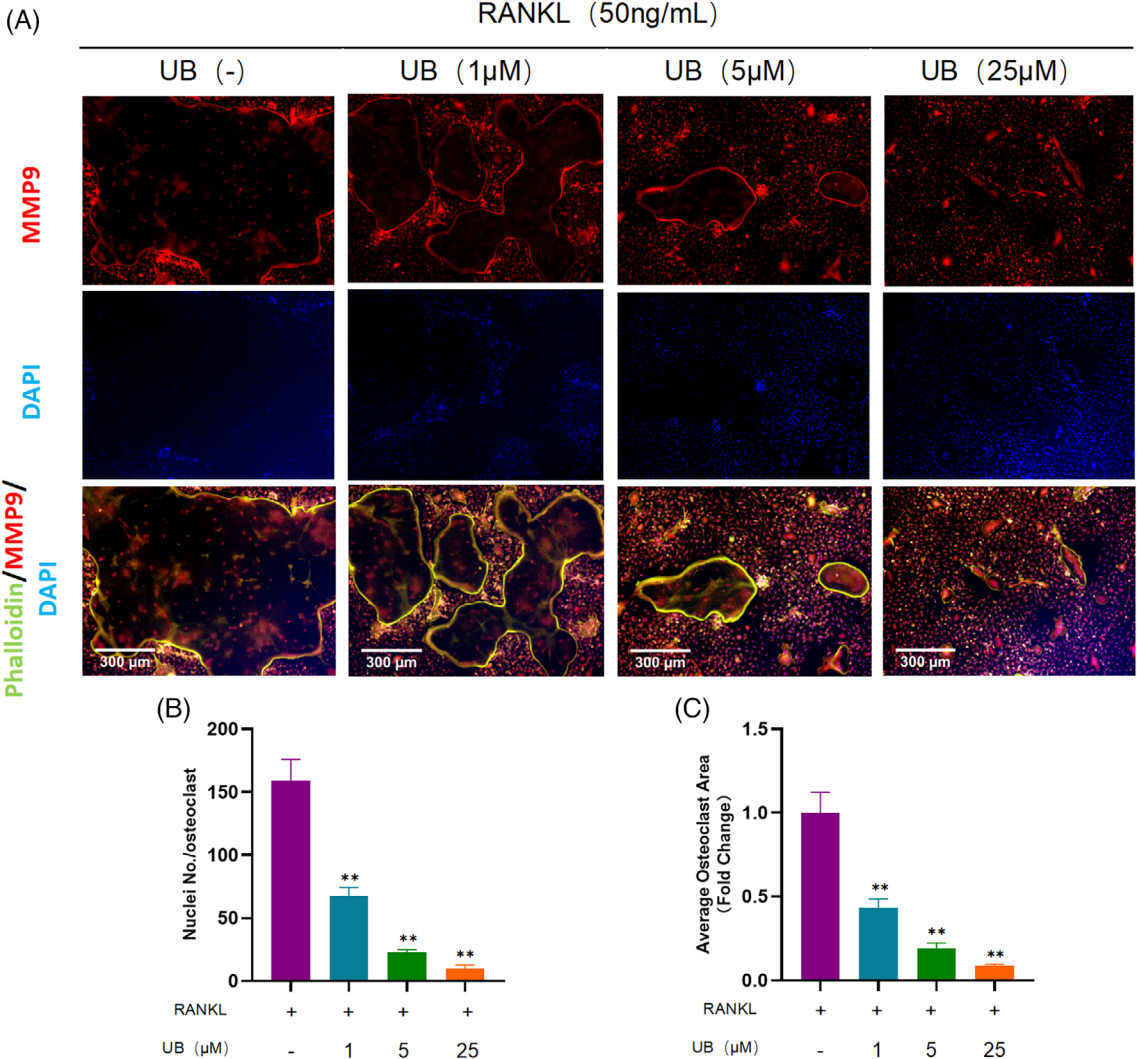
Urolithin B inhibits the formation of ‐ Actin loop in vitro. (A) Representative images of osteoclast related functional protein MMP9, Actin ring and nuclear staining. (B‐C) quantification of the number of nuclei/osteoclasts and the average osteoclast area. *N* = 3 per group; Scale = 300 μm； **p < 0.01 versus the RANKL‐induced group (without UB treatment)

As shown in Figure 1B,C, the cell viability was significantly affected when the concentration of UB was greater than 50 μM via the CCK8 assay. This is consistent with previous research results.^27^ To investigate the function of UB on intervention of osteoclasts differentiation, different concentration of UB, including 0, 1, 5 and 25 μM, was applied to RANKL‐induced BMMs. The TRAP staining results showed that the area of osteoclasts in the UB intervention group was significantly smaller than that in the control group in a concentration‐dependent manner (Figure 1D,E). It is also found by the immunofluorescence staining that the formation of multinucleated osteoclasts was remarkably suppressed by UB intervention (Figure 2A). The actin ring in the RANKL group displayed a huge typical ring structure, while the that in the high UB concentration group was obviously smaller. And the number of nuclei in the ring was significantly less than that in the RANKL group (Figure 2B,C).

### 3.2 UB suppressed the bone resorption function of osteoclasts

**FIGURE 3:**
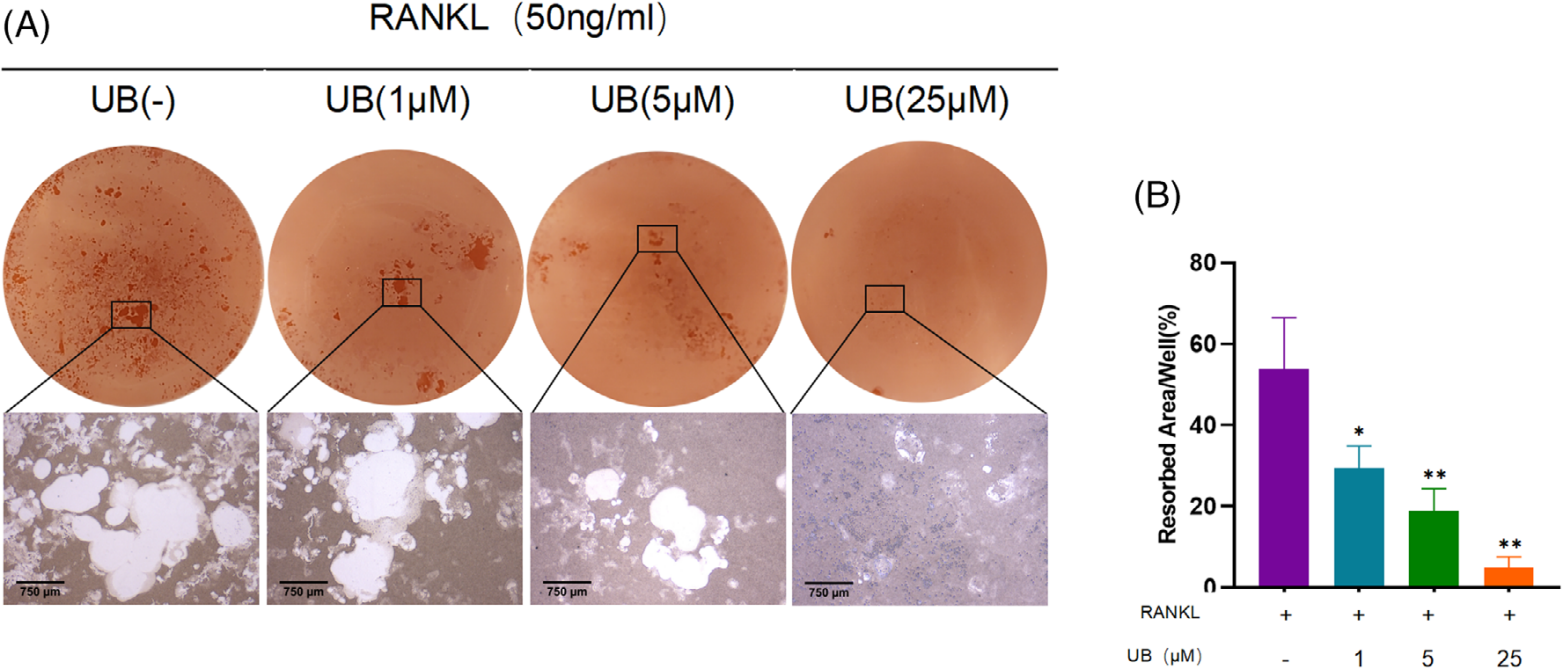
Urolithin B inhibits the bone resorption activity of osteoclasts in vitro. (A) Typical images of bone resorption. (B)Hydroxyapatite resorption assay analysis shows the total resorbed hydroxyapatite area in the BMMs treated with 50 ng/mL M‐CSF, 50 ng/mL RANKL and 0, 1, 5 or 25 μM UB. *n* = 3 per group; Scale = 750 μm；*p < 0.05 ，**p < 0.01 versus the RANKL‐induced group (without UB treatment)

The results of the bone plate resorption assay in the figure revealed that UB significantly inhibited the formation of bone resorption pits mediated by osteoclasts(Figure 3A), and the relative percentages of bone resorption pit areas in the RANKL group and the UB (0, 1, 5 and 25 μM) intervention group were respectively 51.25% ± 8.07%, 29.44% ± 5.39%, 18.83% ± 5.52% and 4.84% ± 2.70%(Figure 3B), indicating that UB treatment showed a dose‐dependent inhibitory effect on the bone resorption function of osteoclasts in vitro.

### 3.3 UB down‐regulated the expression of osteoclast‐related genes and proteins

**FIGURE 4:**
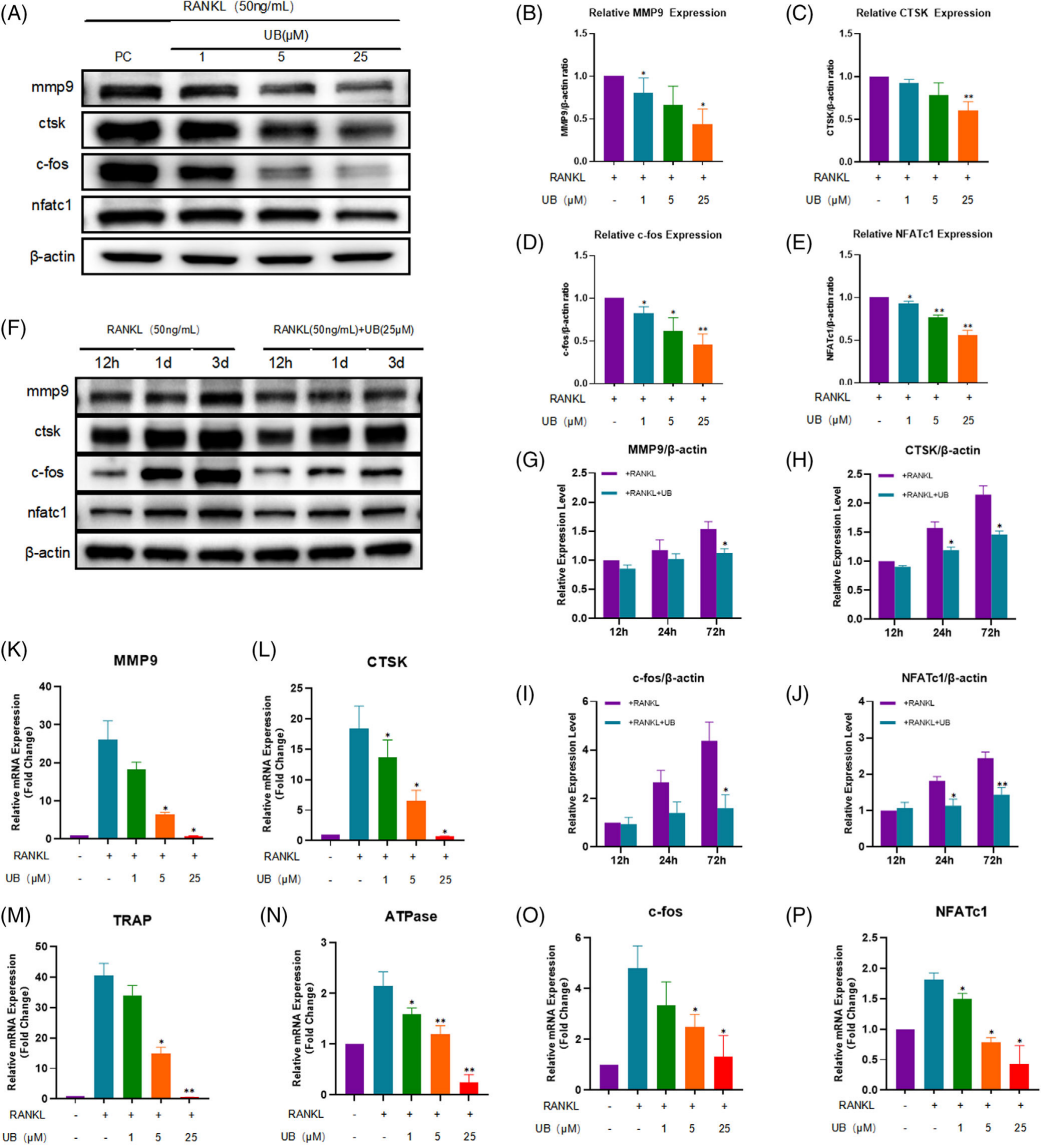
Urolithin B inhibits the expression of osteoclast related proteins and genes. (A、F) Cell lysates were immunoblotted with antibodies against osteoclast associated proteins MMP9, CTSK, c‐fos and NFATc1. (B‐E) Quantitative analysis of concentration dependence of osteoclast associated protein. (G‐J) Quantitative analysis of time‐dependent expression of osteoclast associated protein. *p < 0.05, **p < 0.01 versus the RANKL‐induced group (without UB treatment). (K‐P) Quantitative analysis of mRNA expression of MMP9, CTSK, TRAP, ATPase, c‐fos and NFATc1. *p < 0.05, **p < 0.01 versus the RANKL‐induced group (without UB treatment)

The results of western blotting showed that the expressions of osteoclast‐related functional proteins MMP9 and CTSK were down‐regulated in a concentration‐dependent manner after the intervention of UB (Figure 4A, B‐C). Similarly, the protein expressions of transcription factors c‐fos and NFATc1 related to osteoclast differentiation were also obviously repressed (Figure 4A, D‐E). Through the RT‐PCR experiments, we found that the expression of the osteoclast‐related genes in the model group increased significantly 2 days post RANKL intervention compared with the control group, while that decreased in UB‐treated groups and was concentration‐dependent (Figure 4K‐P). Moreover, we also intervened RAW264.7 cells with RANKL or RANKL + UB (25 μM) for 12 h, 1 and 3 d, respectively. The results showed that the inhibitory efficacy of UB on the expression of RANKL‐induced osteoclast‐related proteins increased with the prolongation of time (Figure 4F, G‐J).

### 3.4 UB repressed RANKL‐induced osteoclastogenesis via downregulation of ERK/NF‐κB signalling pathways

**FIGURE 5:**
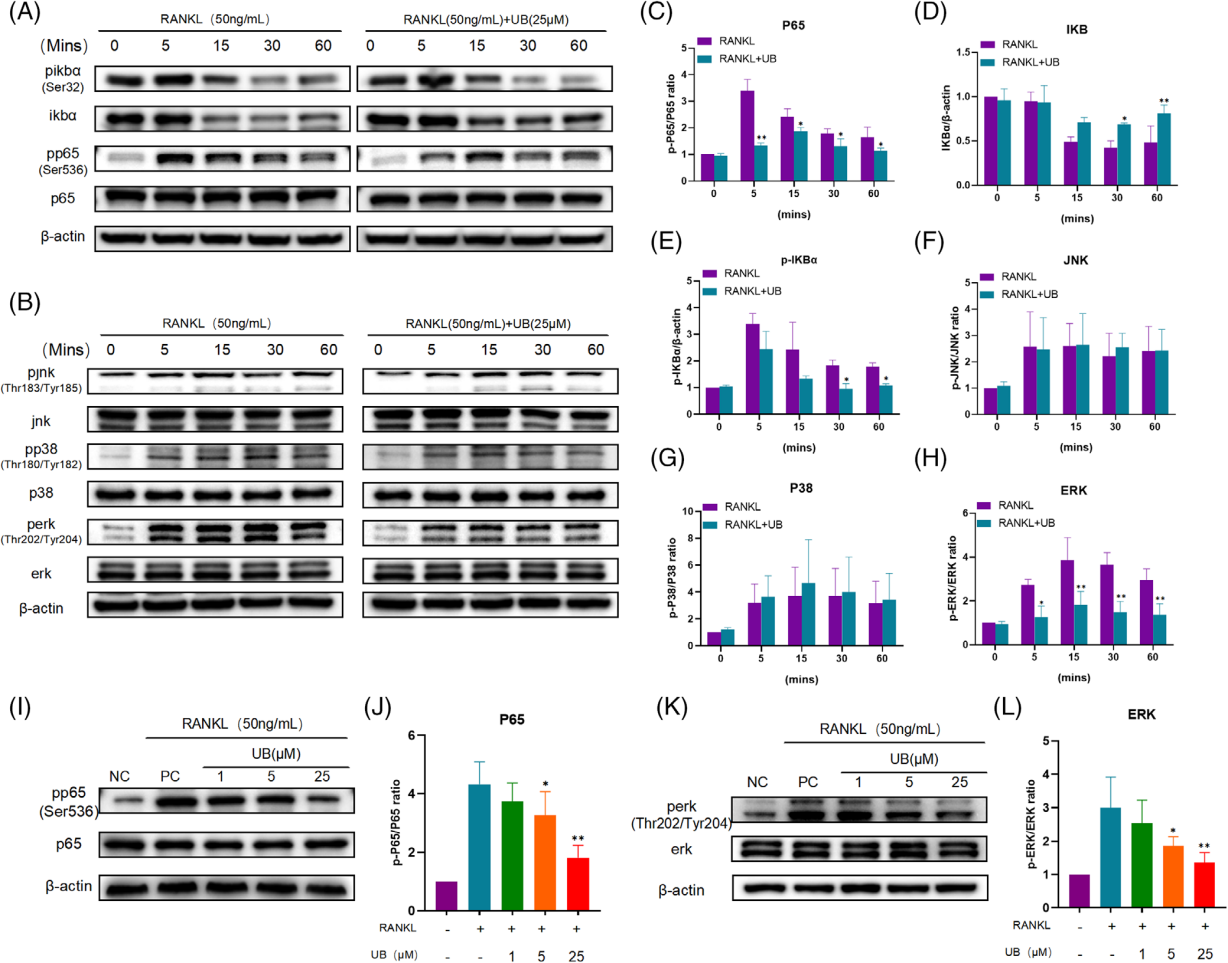
Urolithin B inhibits NF‐κB and ERK signalling pathways inhibit RANKL induced osteoclast differentiation. (A‐B) Typical Western blot images showed the levels of NF‐κB and MAPK pathway proteins in RANKL and RANKL + UB treated raw264.7 cells at 0, 5, 15, 30 and 60 min (C‐H) The histogram shows the relative levels of p‐P65、p‐IκBα、p‐JNK、p‐P38 、p‐ERK proteins in RANKL and RANKL + UB treated raw264.7 cells at 0, 5, 15, 30 and 60 min. *n* = 3 per group, *p < 0.05, **p < 0.01 versus the RANKL induced group at the same time point (I, K) Representative Western blot images showed the corresponding expression levels of p‐P65 and p‐ERK after 30 min of intervention in the blank group, 50 ng/ml RANKL and 0, 1, 5 or 25 μM UB intervention groups. (J, L) Histograms show the corresponding expression levels of p‐P65 and p‐ERK after 30 min of intervention in the blank group, 50 ng/ml RANKL and 0, 1, 5 or 25 μM UB intervention groups. *n* = 3 per group, *p < 0.05, ** *p* < 0.01 versus the RANKL induced group (without UB treatment)

Given that the pathway targets of ellagic acid^16^, ^17^ was previously reported as MAPKs and the NF‐κB pathways (a classical role in osteoclast activation) in inhibition of osteoclast activation, the related proteins in MAPKs, including ERK, JNK and P38, and in NF‐κB pathway were investigated. Results of quantitative analysis revealed that the expression level of phospho‐P65 in the RANKL group increased and reached a peak quickly after RANKL addition, while that in the RANKL + UB group significantly decreased at a parallel time, and the peak period was delayed (Figure 5A, C). Besides, it is displayed that the phosphorylation and degradation of IκBα, an inhibitory protein of NF‐κB, was significantly down‐regulated by UB (Figure 5A, D‐E). Next, the expression of MAPK pathway‐related proteins was evaluated. As shown in Figure, the expression levels of phospho‐ERK, phospho‐JNK and phospho‐p38 of the RANKL‐induced RAW264.7 cells increased with the prolongation of intervention time, while the phospho‐ERK expression in the UB group obviously decreased (Figure 5B, F‐H). In addition, it is observed that the phosphorylation levels of ERK and P65 were down‐regulated with increasing UB concentrations at 30 min post‐treatment (Figure 5I‐L).

**FIGURE 6:**
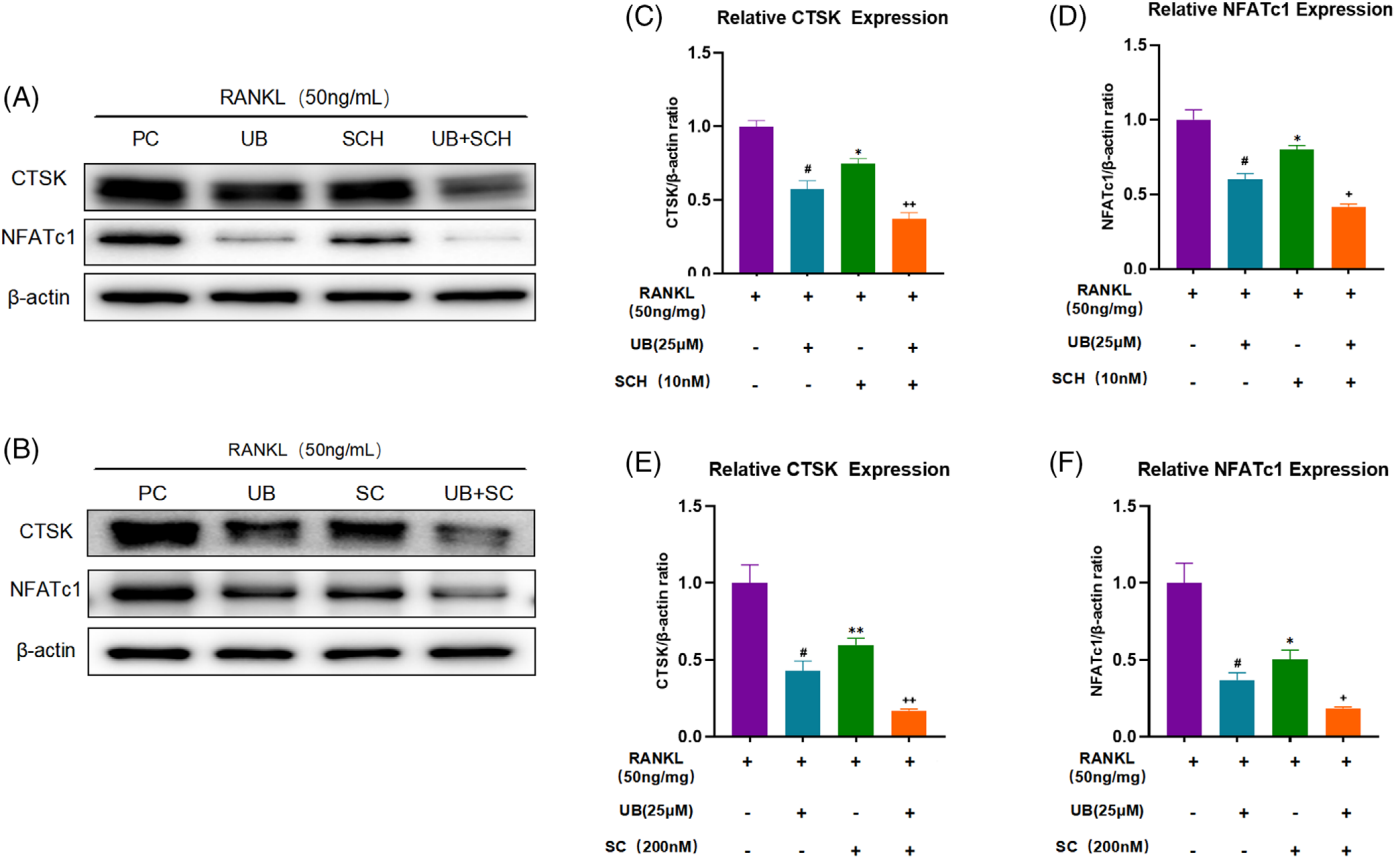
SCH772984 and SC75741 can inhibit the expression of osteoclast‐related proteins. (A‐B) ypical Western blot images of SCH772984 and SC75741 inhibiting osteoclast‐related proteins (C‐F) Histogram showing that SCH772984 and SC75741 inhibit the expression of osteoclast‐related proteins. ^#^ *p* < 0.05 versus the RANKL‐induced group (with SCH treatment), ^+^ *p* < 0.05 、^++^ *p* < 0.01 versus the RANKL‐induced group (with UB treatment)

In following experiments, we used the specific inhibitor SCH772984 to observe the effect of ERK pathway on regulating osteoclasts. The results showed that SCH772984 significantly inhibited the phosphorylation of ERK (Figure S1B‐C). When ERK was specifically inhibited, the expressions of osteoclast‐related proteins CTSK and NFATc1 were significantly down‐regulated, but the effect was minor than that of UB alone. However, the degree of down‐regulation of osteoclast‐related proteins in the SCH772984 + UB intervention group was more significant than that in the UB intervention group (Figure 6A‐D). Next, we used the inhibitor SC75741 to observe the effect of P65 phosphorylation on regulating osteoclasts, and the results showed that SC75741 could significantly downregulate the phosphorylation of P65 (Figure S1E‐F) and reduce the expression levels of osteoclast‐related proteins. The therapeutic effect of UB was more effective than that of SC75741, and the expression of osteoclast‐related proteins was significantly lower than that of SC75741 group and UB group under the simultaneous action of the two drugs (Figure 6B, E,F). Taken together, UB may inhibit osteoclastogenesis through the ERK/NF‐κB signalling pathways.

### 3.5 UB alleviated bone loss in OVX model mice

**FIGURE 7:**
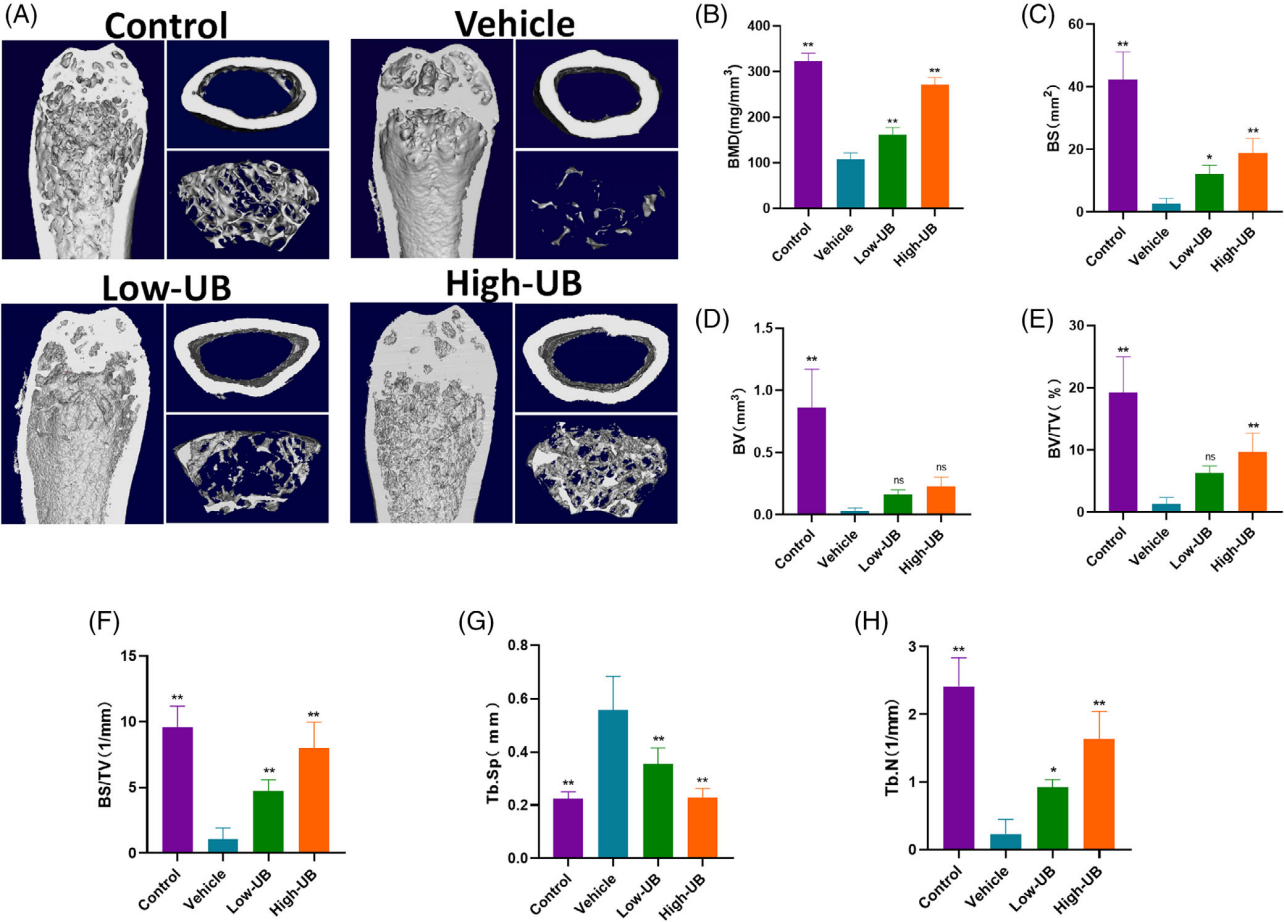
Urolithin B has a protective effect on bone loss in OVX induced osteoporosis model mice. (A) Representative 3D‐μCT images of mouse femurs in sham group, vehicle group and UB treatment group. (B–H) comparative analysis of bone structural parameters, such as bone mineral density (BMD, in g/mm^3^), bone surface area (BS, in mm^2^), bone volume (BV, in mm^3^), ratio of bone volume to total volume (BV/TV, in%), ratio of bone surface to bone volume (BS/BV, in 1/mm), ratio of bone surface to total volume (BS/TV, in 1/mm) Bone trabecular separation (TB. SP, in mm) and number of bone trabeculae (TB. N, in 1/mm). *n* = 5 per group, ** *p* < 0.01 versus the vehicle group

**FIGURE 8:**
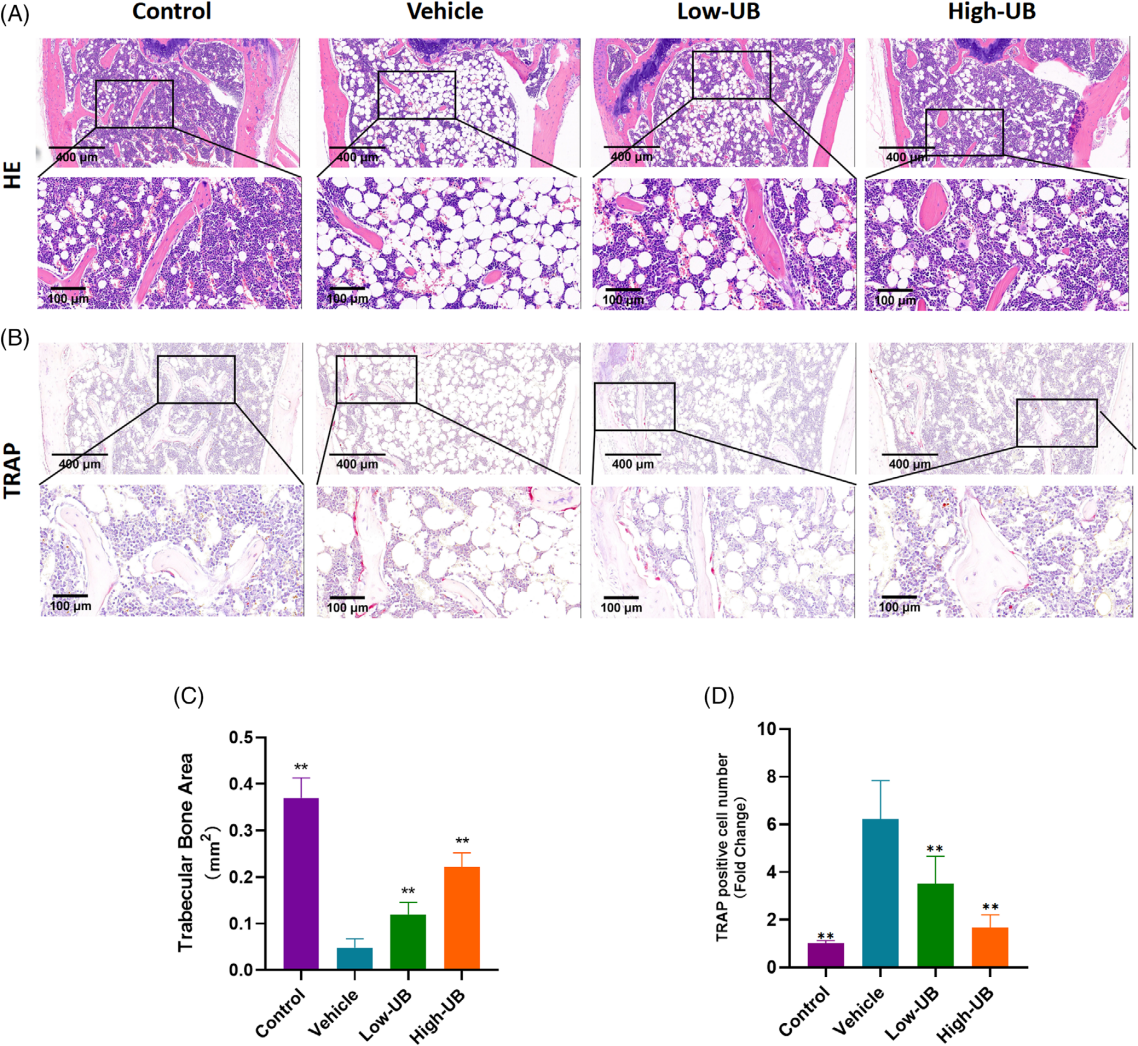
Urolithin B can alleviate bone loss in OVX mice and reduce the number of osteoclasts in bone tissue. (A) Representative H & E staining femoral sections of mice in sham group, vehicle group and UB treatment group (*n* = 5 per group). (B) Representative images showed tartrate resistant acid phosphatase (TRAP) activity staining in bone sections of mice in sham group, vehicle group and UB treatment group. (C) Quantitative analysis of bone trabecular area in femoral sections stained with H & E. (D) The number of trap positive cells in bone sections of sham group, model group and UB treatment group were analysed quantitatively. *n* = 5 per group, ** *p* < 0.01 versus the vehicle group

**FIGURE 9:**
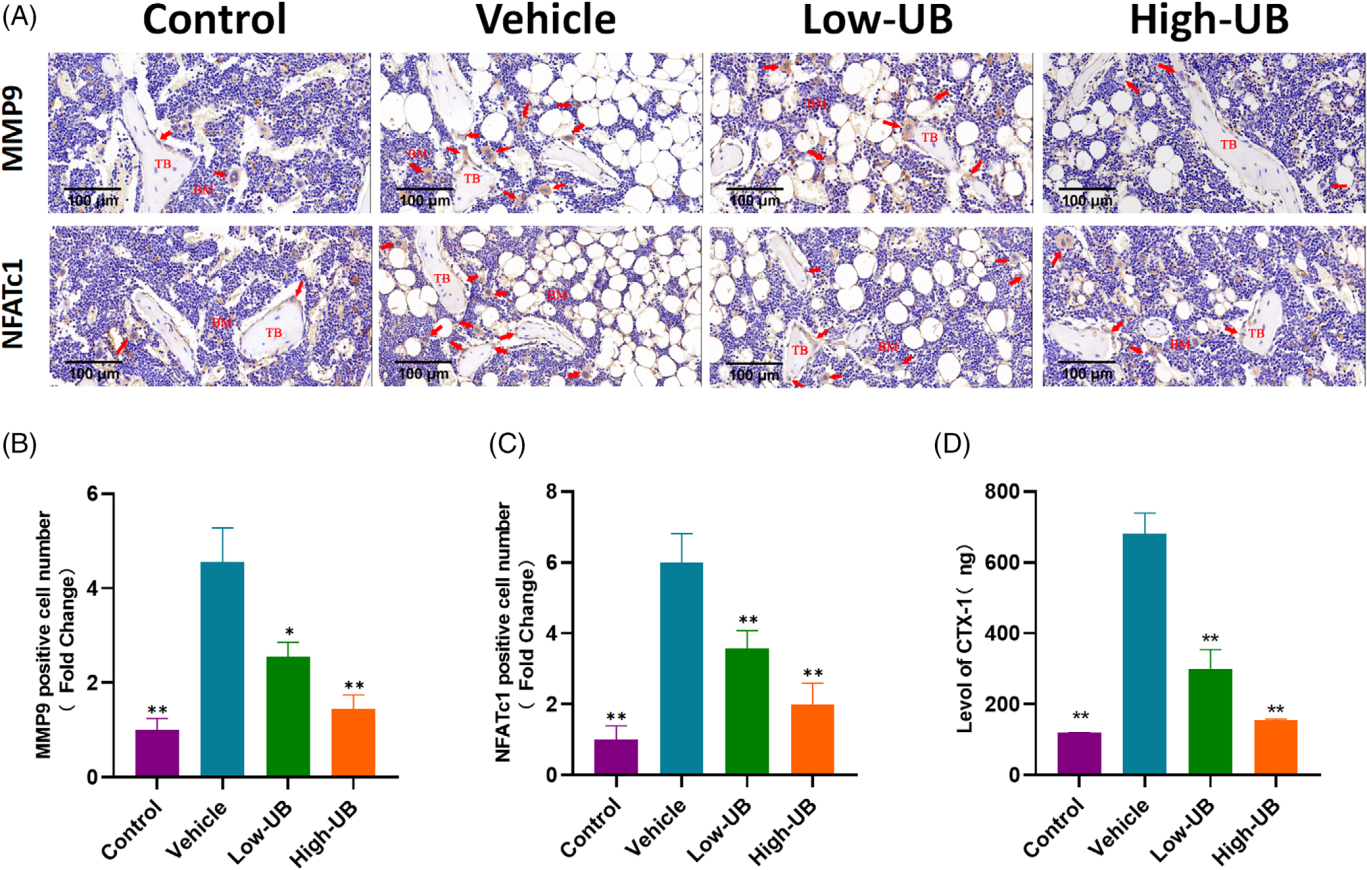
Urolithin B treatment can reduce the number of MMP9 and NFATc1 positive cells in the bone tissue of OVX induced osteoporosis model mice, so as to alleviate the bone loss of OVX mice. (A) Representative images showed IHC staining of osteoclast related proteins MMP9 and NFATc1 in femoral sections of mice in sham group, vehicle group and UB treatment group. (B‐C) the number of MMP9 and NFATc1 positive cells in femoral sections of mice in sham group, vehicle group and UB treatment group were quantitatively analysed. *n* = 5 per group, * *p* < 0.05, * * *p* < 0.01 versus the OVX + vehicle group (D) The content of CTX‐1 in serum of mice in sham group, model group and UB treatment group was analysed quantitatively. *n* = 5 per group, ** *p* < 0.01 versus the vehicle group

To assess the alleviating effect of UB on bone loss in castrated mice, the OVX‐induced osteoporosis mice model was established. At the beginning of modelling establishment and 8 weeks post‐treatment, there was no significant difference in the body weight of the mice in each group, and nearly no damage caused by UB was observed in the liver and kidney slices in the intervention group indicating that UB had almost no cytotoxicity (Figure S2). The structural parameters of distal femurs of mice in sham group, OVX group and OVX + UB group were analysed by micro‐CT. The results showed that the parameters of the distal femur of mice in the OVX group significantly decreased compared to those in the sham group (BMD in g/cm^3^: 0.11 ± 0.01 versus 0.32 ± 0.02, BV in mm^3^: 0.03 ± 0.02 versus 0.86 ± 0.31, BS in mm^2^: 2.53 ± 1.82 versus 42.34 ± 8.79, BV/TV in %: 1.29 ± 1.07 versus 19.22 ± 5.78, BS/TV in 1/mm: 1.06 ± 0.86 versus 9.58 ± 1.60, Tb. Sp in mm: 0.56 ± 0.13 versus 0.22 ± 0.03, Tb. N in 1/mm: 0.23 ± 0.21 versus 2.41 ± 0.02, respectively), while those parameters in the OVX + UB group remarkably increased in a concentration‐dependent manner (Figure 7A‐H).The results of H&E staining revealed that UB treatment decreased OVXinduced bone mass loss (Figure 8A,C). TRAP staining results of bone tissue sections demonstrated that the number of osteoclasts/bone surface (N. Oc/BS) in the OVX group significantly increased compared with those in the sham group, while the UB treatment decreased the N.Oc. /BS parameters depending on the concentration change (Figure 8B,D). Furthermore, the results of immunohistochemistry staining analysis of osteoclast functional protein MMP9 and related transcription factor NFATc1 showed that the amount of MMP9‐ and NFATc1‐ positive cells in the OVX group displayed an obvious increasing tendency compared to the sham group, while decreased in the OVX + UB group (Figure 9A‐C). We also detected and compared the serum bone resorption index CTX‐1 in mice by ELISA. It was shown that the serum CTX‐1 content in the OVX + UB group was significantly lower than that in the OVX group (Figure 9D).

## 4 DISCUSSION

Osteoporosis caused by imbalance of bone homeostasis has become a public health issue that cannot be ignored nowadays, and the abnormal activation of osteoclasts is the key to the progression of osteoporosis. The main targets of traditional osteoporosis therapeutics are osteoclasts, but the long‐term application of such drugs will also cause side effects.^6^, ^7^ Thus, how to more safely and effectively inhibit the abnormal activation of osteoclasts has become an urgent clinical need. Natural compounds like glycyrrhizic acid^28^ and puerarin^29^ are becoming promising candidates for osteoporosis due to their low toxicity and side effects. Similarly, the search for potential drug molecules from the metabolites of intestinal flora has gradually raised wide attentions in recent years.^10^ For example, Dylan Dodd et al.^30^ found that gut symbiont Clostridium sporogenes could generate indolepropionic acid to regulate the systemic immunity by metabolizing aromatic amino acids. Some researchers have reported that the intestinal flora metabolite N‐acylamide was a ligand analog of G protein‐coupled receptors and can modulate glucose homeostasis.^9^ Given that ellagitannin, the precursor of UB, has been reported to alleviate osteoporosis in castrated mice,^18^, ^19^ and UB is more bioavailable,^21^ we explored the effect of UB on osteoclasts and its mechanism of action.

Osteoclasts are the cells responsible for bone resorption, which could be divided into steps, including bone adsorption, cytoskeleton reorganization, and vesicle trafficking.^31^ When osteoclasts adhere to the bone surface, the actin ring formed by cytoskeleton reorganization^32^ is tightly connected with the bone surface and constitutes an acidic bone resorption microenvironment.^33^ Next, osteoclasts transport osteoclast‐related functional proteins such as CTSK, MMP9, etc. through vesicles to the acidic absorption microenvironment, and then internalize bone degradation products through vesicles.^34^ Therefore, it can be seen that the number and area of osteoclasts attached to the bone surface and the secretion of osteoclast‐related functional proteins are equally vital for the bone resorption. In this study, we found that UB could inhibit the formation of RANKL‐induced osteoclasts in a concentration‐dependent manner, and the number and area of osteoclasts were significantly reduced with the increase of UB concentration by TRAP staining. Also, the formation of the osteoclast actin ring was clearly suppressed by UB via the immunofluorescence staining, and the fluorescent signal of the functional protein MMP9 was also weakened due to UB intervention. The results of WB and PCR experiments were also consistent with the above studies, showing that the expression of related functional proteins decreased related with UB concentration. Furthermore, the bone plate resorption test also directly indicated that UB repressed the osteoclast activity. Taken together, our results demonstrated that UB held an inhibitory effect on the formation and activation of osteoclasts leading to the reduction of bone resorption activity in vitro.

The activation of NF‐κB and MAPK pathways is crucial for the maturation and activation of osteoclasts.^35^ XU et al. proved that ellagitannin and ellagic acid can inhibit the formation of osteoclasts through the above two classical pathways,^36^ while Lee et al. figured out that UB could down‐regulate the phosphorylation of ERK protein in LPS‐stimulated microglia^13^ and the nuclear import of P65 in RAW264.7 cells,^27^ thereby exerting an anti‐inflammatory mechanism. In this study, results of western blotting assay showed that UB could inhibit the phosphorylation and degradation of IκBα, also the phosphorylation of p65. IκBα is a typical member of the IκB family with typical features of IκB proteins. The typical p65/p50 heterodimer is mostly bound to IκBα and is in an inactive state, and rapid signalling‐induced phosphorylation and subsequent degradation of IκBα protein is required for NF‐κB p65 nuclear localization and DNA binding.^37^ In addition, we also observed that UB can suppress the phosphorylation of ERK protein in the MAPK pathway and the expression of downstream osteoclast‐related transcription factors NFATc1 and c‐fos in a dose‐dependent manner. Various upstream stimulators in the ERK pathway positively regulate the differentiation of osteoclasts.^38^ Various drugs and natural compounds, such as bisphosphonates^39^ and hypericin,^40^ have been reported to play a role in inhibition of osteoclastogenesis via down‐regulation of the ERK signalling pathway.^38^ In the observation of MAPK pathway by UB, the phosphorylation level of ERK reached a peak at 15 min after RANKL intervention, and that was significantly down‐regulated after treatment with UB.

The increased activity of osteoclasts caused by hypoestrogenism can lead to rapid bone loss.^41^ In this study, the inhibitory effect of UB on bone resorption was verified in vivo by ovariectomy‐induced mouse osteoporosis model. In vivo results observed a significant decrease in distal femur bone mineral density in model group when compared to blank group, indicating the success in OVX osteoporosis mouse model construction. Next, the morphological and histological observations of the distal femur confirmed that the formation and activation of osteoclasts were inhibited by UB. Similarly, LIN et al. verified that intraperitoneal injection of ellagic acid can alleviate ovariectomy‐induced osteoporosis in mice.^19^ However, some scholars believe that the bioavailability of ellagic acid is low, and future studies should use its derivative, UB, which is more bioavailable to investigate its potential efficacy.^21^ UB has been confirmed to have a high bioavailability in vivo, and can reach therapeutic concentration for benign intervention in skeletal muscle,^42^ leukocytes,^43^ and cardiomyocytes^44^ in vivo. In this study OVX bone loss was significantly alleviated in mice treated with UB and the content of CTX‐1, a specific marker of bone resorption in serum, was also significantly reduced. Besides, no drug toxicity was observed in liver and kidney sections. Therefore, UB can be considered as a safe and effective treatment in inhibiting osteoclast activity in vivo.

Overall, this study also has some limitations. Although we have verified that UB can inhibit osteoclast activation and alleviate osteoporosis by in vivo and in vitro experiments. However, osteoclast activation involves autoimmunity,^45^, ^46^ inflammation,^47^ oxidative stress^48^ and other factors, and UB and ellagic acid have been reported to have anti‐inflammatory and antioxidant effects.^49^ Therefore, whether the regulated osteoclasts are attributed to the anti‐inflammatory and antioxidant effects of UB should be further studied.

## 5 CONCLUSION

**SCHEME 1:**
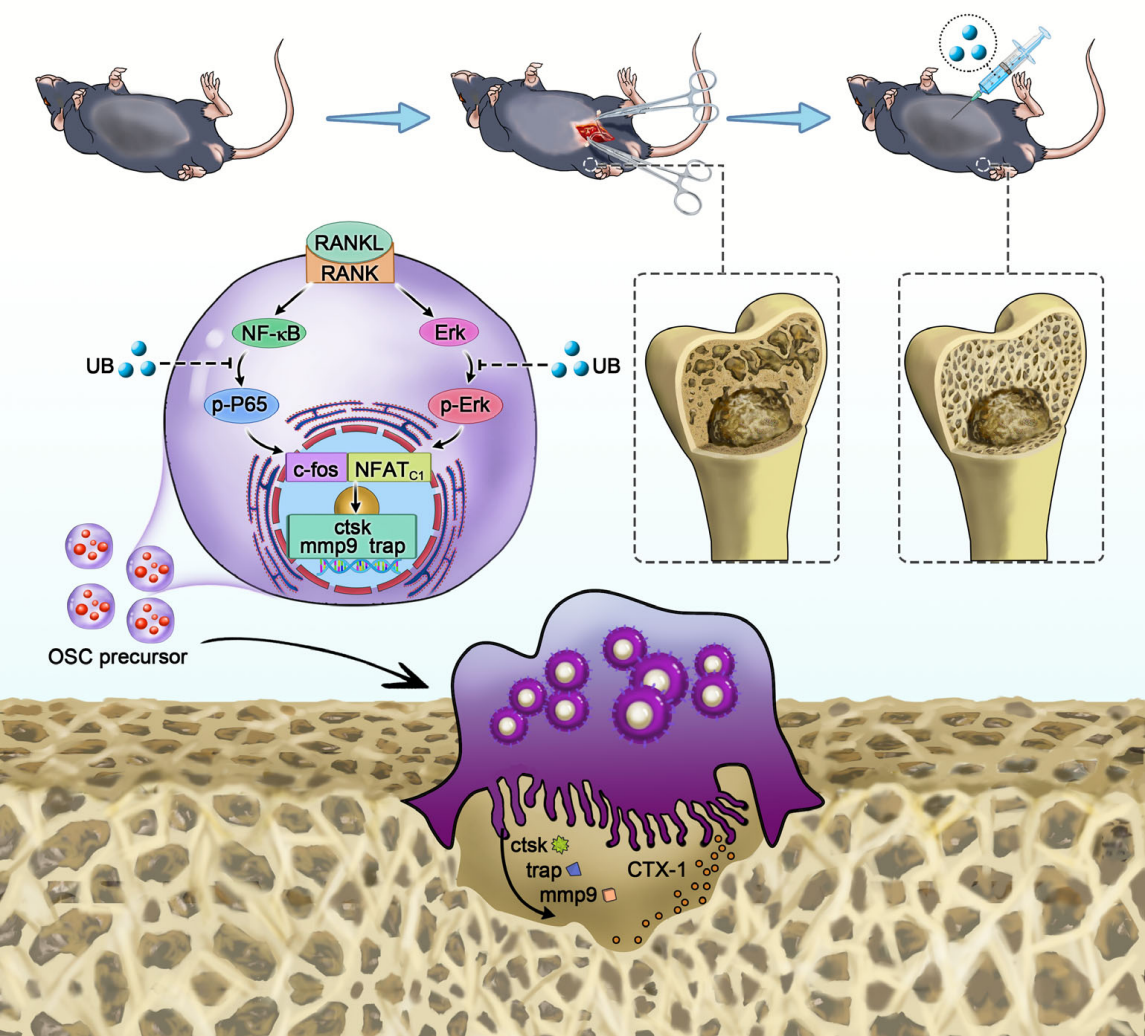
Urolithin B can alleviate bone loss in ovariectomized mice in vivo and inhibit the formation and activation of RANKL induced osteoclasts in vitro. As shown in the figure, urolithin B inhibits the activation of downstream transcription factors NFATc1 and c‐fos by down regulating the phosphorylation levels of p65 and ERK and reduces the expression of osteoclast related functional proteins CTSK, TRAP and MMP9 to inhibit the formation and activation of osteoclasts.

Overall, the in vitro studies demonstrated that UB could decrease osteoclast activation by inhibiting the ERK/NF‐κB signalling pathway (Scheme 1). In vivo experiments showed that UB could alleviate the progression of osteoporosis in OVX mice (Scheme 1). Based on these findings, we believe that UB is a safe and effective and can be considered as a potential treatment for osteoporosis.

## AUTHOR CONTRIBUTIONS

Zhirong Wang, Dechun Geng and Long Xiao are corresponding authors who conceptualized, designed, and supervised the research project, and revised the manuscript. Yajun Li, Qi Zhuang, Kai Zheng and Lihong Tao designed and performed in vivo and in vitro experiments, data analysis, manuscript writing and revisions. Shuangshuang Chen, Qi Zhuang and Yunshang Yang performed micro‐CT, IHC staining assays, data analysis and helped with the experimental methods and Figures. Chengcheng Feng, Zhifang Wang and Haiwei Shi contributed to the in vivo experiments and helped with the experimental methods. Jiandong Shi and Yiling Fang performed the figures. All authors read and approved the final manuscript.

## CONFLICT OF INTEREST

The authors have declared that no competing interest exists.

## CONSENT FOR PUBLICATION

This manuscript does not contain any individual person's data in any form. Consent for publication is not applicable.

## Supporting information


**Figure S1** (A) CCK8 assay was used to evaluate the cytotoxicity of SCH772984. (B) Typical western blot images show that SCH772984 specifically inhibit the phosphorylation of ERK. (C) The histograms show the corresponding expression levels of p‐ERK in the 50 ng/mL RANKL and inhibitor intervention groups after 30 min of intervention. Compared with RANKL induction group, *n* = 3 in each group, **p < 0.01. (D) CCK8 assay was used to evaluate the cytotoxicity of SC75741. (E) Typical western blot images show that SCH772984 specifically inhibit the phosphorylation of P65. (F) The histograms show the corresponding expression levels of p‐p65 in the 50 ng/mL RANKL and inhibitor intervention groups after 30 min of intervention. Compared with RANKL induction group, *n* = 3 in each group, **p < 0.01

**Figure S2** (A) H & E staining of liver and kidney tissue sections of OVX induced osteoporosis model mice treated with urolithin B

## Data Availability

The date used to support the findings of this study are available from the corresponding author upon request.
