# Supplementary figures and images for: Urolithin B suppressed osteoclast activation and reduced bone loss of osteoporosis via inhibiting ERK/NF‐κB pathway
Source: Cell Prolif. 2022 Jun 16;55(10):e13291. doi: 10.1111/cpr.13291 (PMC9528769; doi:10.1111/cpr.13291)

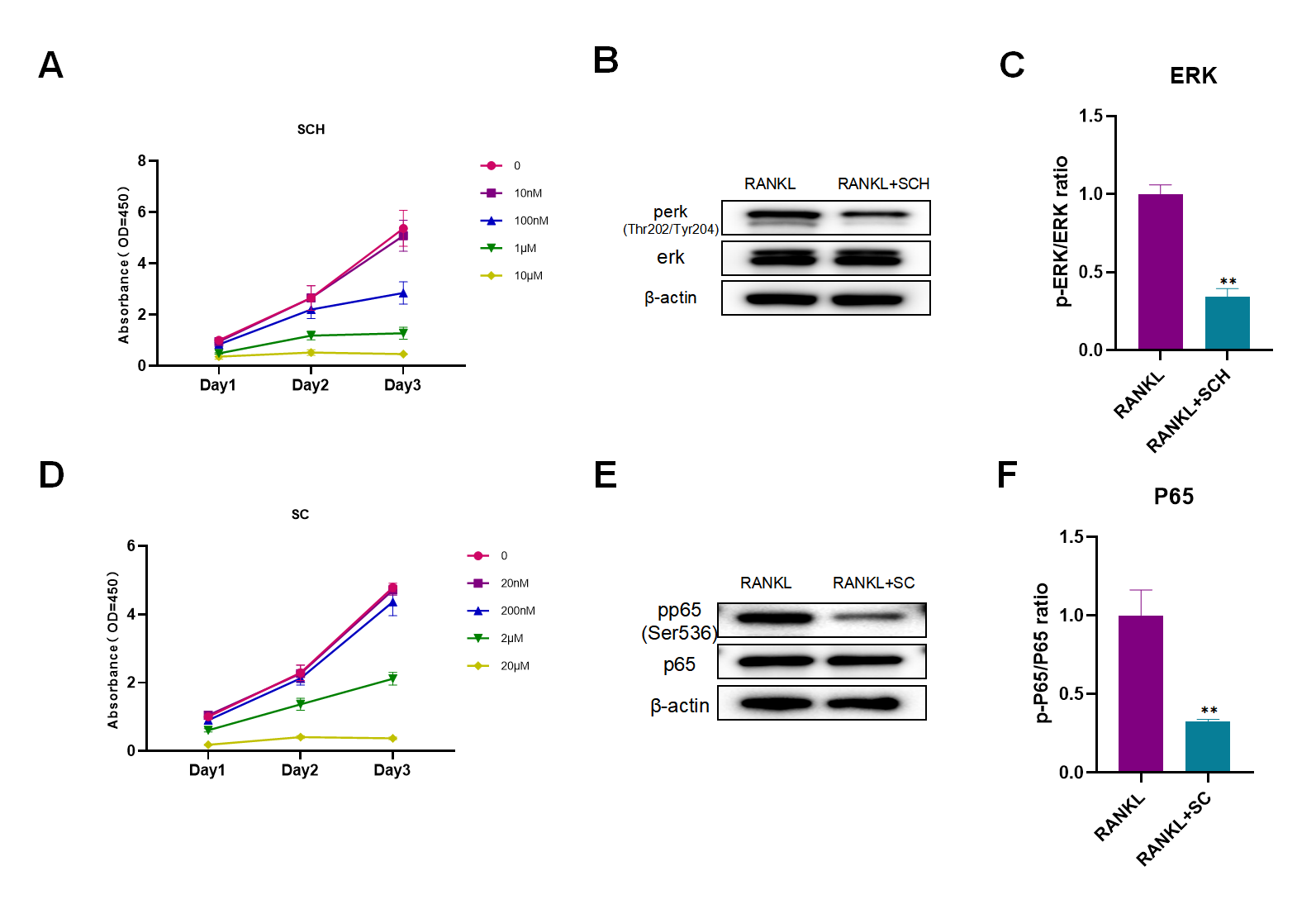

Supplement: Supplementary file 1 — Figure S1 (A) CCK8 assay was used to evaluate the cytotoxicity of SCH772984. (B) Typical western blot images show that SCH772984 specifically inhibit the phosphorylation of ERK. (C) The histograms show the corresponding expression levels of p‐ERK in the 50 ng/mL RANKL and inhibitor intervention groups after 30 min of intervention. Compared with RANKL induction group, n = 3 in each group, **p < 0.01. (D) CCK8 assay was used to evaluate the cytotoxicity of SC75741. (E) Typical western blot images show that SCH772984 specifically inhibit the phosphorylation of P65. (F) The histograms show the corresponding expression levels of p‐p65 in the 50 ng/mL RANKL and inhibitor intervention groups after 30 min of intervention. Compared with RANKL induction group, n = 3 in each group, **p < 0.01 [file CPR-55-e13291-s001.tif]

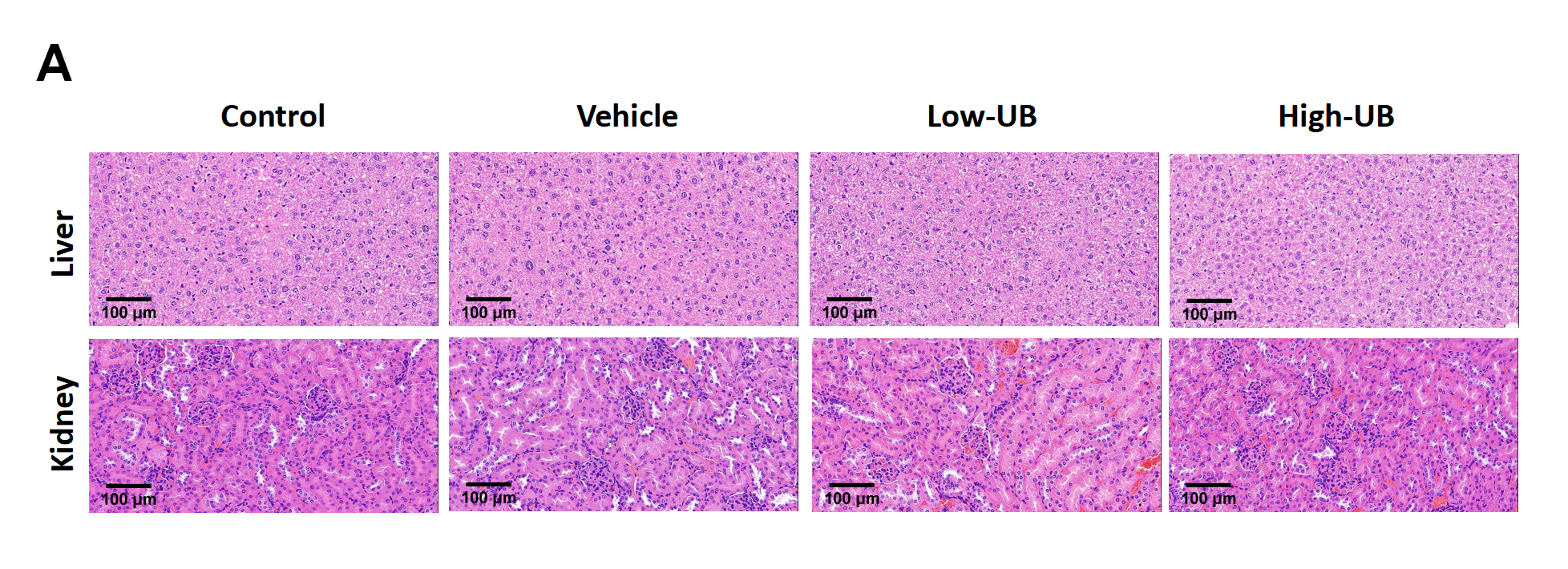

Supplement: Supplementary file 2 — Figure S2 (A) H & E staining of liver and kidney tissue sections of OVX induced osteoporosis model mice treated with urolithin B [file CPR-55-e13291-s002.tif]
